# *XAF1 *expression and regulatory effects of somatostatin on *XAF1 *in prostate cancer cells

**DOI:** 10.1186/1756-9966-29-162

**Published:** 2010-12-11

**Authors:** Zhaoquan Xing, Zunlin Zhou, Rong Yu, Shuling Li, Chunde Li, Sten Nilsson, Zhaoxu Liu

**Affiliations:** 1 Department of Integrated Traditional Chinese and Western Medicine, Qilu Hospital, Shandong University, Jinan, 250012 P.R. China; 2 Department of Urology, Qilu Hospital, Shandong University, Jinan, 250012 P.R. China; 3 Ageing Center, School of Nursing, Shandong University, Jinan, 250012 P.R. China; 4 Department of Pathology & Oncology, Karolinska Institutet, 171 76 Stockholm, Sweden

## Abstract

**Background:**

Somatostatin prevents cell proliferation by inducing apoptosis. Downregulation of the *XAF1 *transcript may occur during the development of prostate cancer. It is interesting to evaluate the potential regulatory effects of somatostatin on *XAF1 *expression during the development of prostate cancer cells.

**Methods:**

*XAF1 *mRNA and protein expression in human prostate epithelial cells RWPE-1, androgen dependent prostate cancer LNCaP, and androgen independent DU145 and PC3 cells were evaluated using RT-PCR and Western blot. The regulation of *XAF1 *mRNA and protein expression by somatostatin and its analogue Octreotide was evaluated.

**Results:**

Substantial levels of *XAF1 *mRNA and proteins were detected in RWPE-1 cells, whereas prostate cancer cells LNCaP, DU145 and PC3 exhibited lower *XAF1 *expression. Somatostatin and Octreotide up-regulated *XAF1 *mRNA and protein expression in all prostate cancer cell lines.

**Conclusions:**

*XAF1 *down-regulation may contribute to the prostate cancer development. The enhanced *XAF1 *expression by somatostatin indicates a promising strategy for prostate cancer therapy.

## Background

Prostate cancer is the most common cancer and the leading cause of cancer death among men in the United States and Europe [1,2]. It was estimated that approximately 186,320 new cases and 28,660 prostate cancer-related deaths occurred in the US in 2008 [1]. Although epidemiological studies showed that the incidence of prostate cancer in Asians is much lower than that in African-Americans [3], the occurrence of the disease has rapidly increasing in China[4]. Most prostate cancers are initially androgen-dependent but become androgen-independent and refractory to hormone withdrawal therapy [5]. Like all other human malignancies, prostate cancer cells escape apoptotic death through highly efficient pathways involving multiple mechanisms [6,7].

X-linked inhibitor of apoptosis protein-associated factor-1 (*XAF1*) was first identified as an interacting protein of X-linked inhibitor of apoptosis (*XIAP*) [8]. *XIAP *suppresses apoptotic cell death by binding to caspases and inhibiting their functions. *XAF1 *antagonizes *XIAP *activities, thereby promoting apoptosis [9]. *XAF1 *can dramatically sensitize cancer cells to apoptotic triggers such as TRAIL, etoposide treatments 5-fluorouracil [10], H_2_O_2_, c-irradiation, ultraviolet [11], and tumour necrosis factor-α, which are independent of its interaction with *XIAP *[12]. *XAF1 *is therefore believed to play an important role in the major apoptosis-related pathways. *XAF1 *also serves as a candidate tumour suppressor gene. Loss of *XAF1 *has been observed in a variety of cancer cell lines and human cancers [13-16]. However, little is yet known about its potential implication in prostate cancer.

So far, there have been no effective therapeutic measures for the treatment of hormone refractory prostate cancer. Treatment with somatostatin may therefore be a possible therapeutic alternative to chemotherapy in hormone refractory prostate cancer patients. Somatostatin, originally identified as a neuropeptide inhibiting growth hormone release more than 30 years ago, is widely present in central and peripheral human cells/tissues including prostate. Somatostatin has been shown to exert a potent anti-tumour action by affecting tumour cell proliferation, apoptosis, angiogenesis and the host's immune response [17-21]. Octreotide is an analogue of somatostatin and has been used in clinical practice since data emerged in the 1980 s confirming its ability to palliate carcinoid syndrome [22]. Our previous results have shown that somatostatin may affect the mitochondria of LNCaP and DU145 cells in a way that eventually triggers mitochondrial-mediated apoptosis and exert its effects on prostate cancer cells via MAPK pathway and by regulating the activities of phosphotyrosine phosphatases [23].

In the current study, we examined *XAF1 *mRNA and protein expression in four cell lines, and determined regulatory effects of somatostatin and Octreotide on *XAF1 *expression in prostate cancer cell lines. We found that somatostatin and Octreotide up-regulated *XAF1 *mRNA and protein expression in prostate cancer cell lines. The enhanced *XAF1 *expression by somatostatin indicates a promising strategy for prostate cancer therapy.

## Materials and methods

### Cell lines and cell culture

A human prostate epithelial cell line (RWPE-1) and prostate cancer cell lines (LNCaP, DU145 and PC3) were used and were obtained from the American Type Culture Collection (ATCC). LNCaP, DU145 and PC3 were maintained in RPMI-1640 medium supplemented with 10% foetal bovine serum (FBS). RWPE-1 cells were maintained in complete keratinocyte serum-free medium (K-SFM) containing 50 μg/ml bovine pituitary extract and 5 ng/ml epidermal growth factor. The cultures were maintained in a humidified 5% CO_2 _environment at 37°C. The medium was changed twice a week and the cells were trypsinized and subcultivated once a week. Somatostatin and Octreotide (Sigma) were prepared as described previously [24]. The cells were treated with 1 nM somatostatin and 1 nM Octreotide for different periods of time (0, 1 h, 12 h, 24 h, 72 h), as described by Brevini [25]. Controls were untreated cells.

### RNA extraction and RT-PCR

*XAF1 *mRNA was detected using reverse transcription PCR (RT-PCR). Total cellular RNA was extracted using Trizol reagent (Invitrogen, Carlsbad, CA), according to the manufactures' instruction. cDNA was synthesized using random primers (N6) and M-MLV reverse transcriptase. PCR was performed by using *XAF1*-specific primers as follows: forward: 5'-ATG GAA GGA GAC TTC TCG GT-3'; reverse: 5'-TTG CTG AGC TGC ATG TCC AG-3' and the conditions were: denaturation at 94°C for 5 min, followed by 34 cycles of 94°C 30 s, 60°C 30 s, 72°C 45 s, and then a final cycle of 10 min at 72°C. Amplification products (290 bps) were electrophoresed onto 1.5% agarose gels and visualized by 0.5% ethidium bromide staining. The results of electrophoresis were analyzed by the Gel Image System Fluor Chem TM 9900 (Alpha Innotech).

### Western blot analysis

Cells were lysed in buffer containing 50 mM Tris-HCl (pH 7.5), 250 mM NaCl, 0.1% NP-40 and 5 mM EGTA, 50 mM sodium flu-oride, 60 mM β-glycerol-phosphate, 0.5 mM sodium-vanadate, 0.1 mM PMSF, 10 μg/ml aprotinin and 10 μg/ml leupeptin. Protein concentration was determined using the BCA protein assay kit (Pierce Bio-technology, Inc., USA). Protein samples (40 μg) were subjected to a 10% SDS-PAGE and electrophoretically transferred to PVDF membranes (Bio-Rad, Hercules, CA, USA). The membranes were first incubated with 5% nonfat milk in Tris-buffered saline (TBS). After washing three times in 0.1% Tween 20-TBS (TBST), the membranes were incubated with primary antibody (goat anti-human *XAF1*, 1:600; Santa Cruz Biotecnology) and β-actin (rabbit anti-actin antibody R-22, 1:1000; Santa Cruz Biotecnology) separately at 4°C overnight, followed with the corresponding secondary antibodies separately (1:2500) for 1.5 h at room temperature and the antibody-bound proteins were detected by the ECL system (Amersham Biosciences, Little Chalfont Buckinghamshire, UK).

## Results

### Expression of *XAF1 *mRNA and protein in prostate cell lines

**Figure 1:**
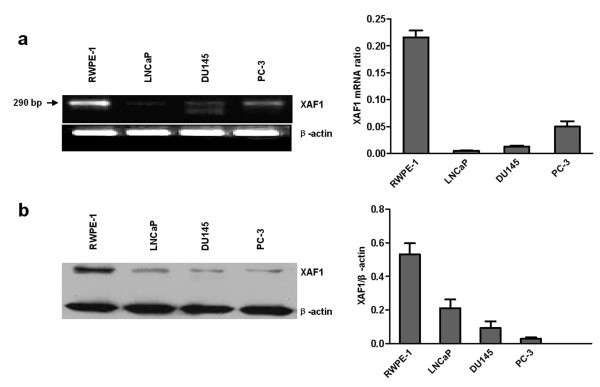
**Expression of *XAF1 *mRNA and protein in human prostate cell lines**. a. RT-PCR analysis of *XAF1 *mRNA; the β-actin transcript was analyzed as a control. b. Western blot analysis of *XAF1 *protein; the β-actin was as a control.

The expression of *XAF1 *was detected at mRNA and protein levels with RT-PCR and Western blot. As shown in Figure 1, RT-PCR using cDNA primers specific for a segment of the human *XAF1 *mRNA provided a product of the expected size in four prostate cell lines. It showed lower expression of *XAF1 *mRNA in prostate cancer cells LNCaP, DU145 and PC3 compared with that in RWPE-1 cells which displayed the strongest expression of *XAF1 *mRNA among all four cell lines. We found protein expression of *XAF1 *in these same cell lines by Western blot analysis, consistent with *XAF1 *mRNA expression. (Figure 1).

### Up-regulation of *XAF1 *mRNA and protein by somatostatin and Octreotide in prostate cancer cell lines

**Figure 2:**
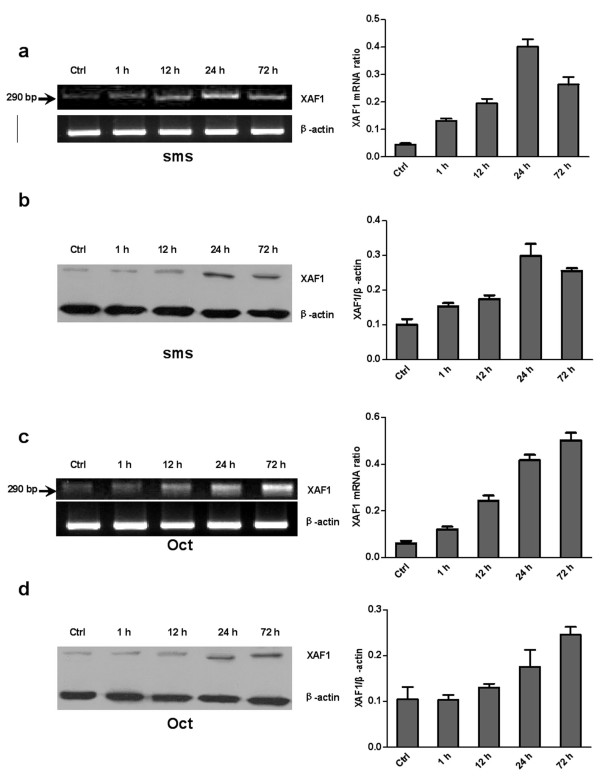
**Time-dependent somatostatin and Octreotide-induced expression of *XAF1 *mRNA and protein in LNCaP cell line**. Cells were stimulated with 1 nM somatostatin (a and b) and 1 nM Octreotide (c and d) for the time periods indicated. a and c: RT-PCR results. b and d: Western blot. Oct: Octreotide; sms: somatostatin.

**Figure 3:**
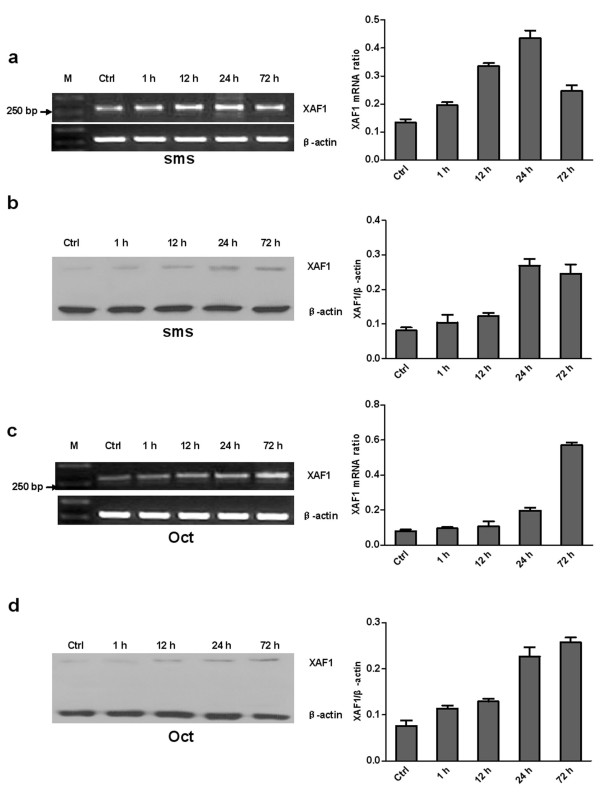
**Time-dependent somatostatin and Octreotide-induced expression of *XAF1 *mRNA and protein in DU145 cell line**. Cells were stimulated with 1 nM somatostatin (a and b) and 1 nM Octreotide (c and d) for the time periods indicated. a and c: RT-PCR results. b and d: Western blot. Oct: Octreotide; sms: somatostatin.

**Figure 4:**
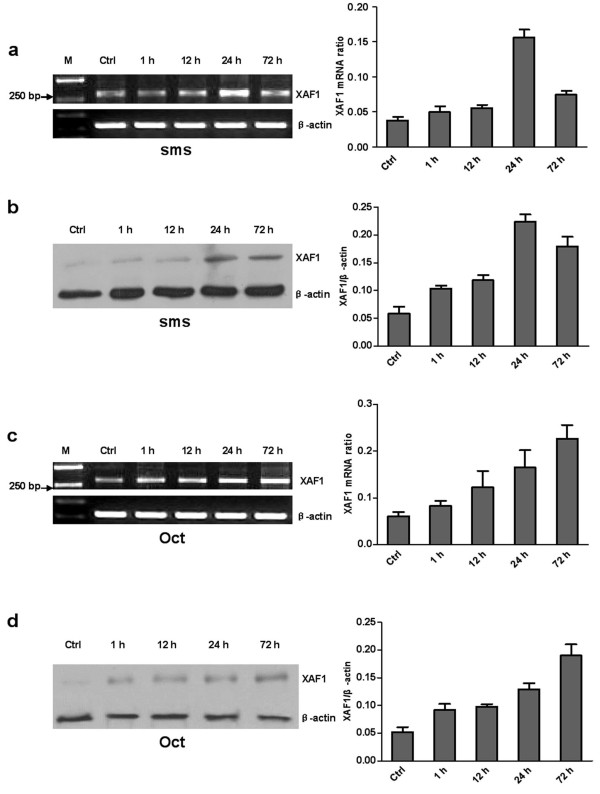
**Time-dependent somatostatin and Octreotide-induced expression of *XAF1 *mRNA and protein in PC3 cell line**. Cells were stimulated with 1 nM somatostatin (a and b) and 1 nM Octreotide (c and d) for the time periods indicated. a and c: RT-PCR results. b and d: Western blot. Oct: Octreotide; sms: somatostatin.

To examine the regulatory effects of somatostatin and Octreotide on *XAF1 *mRNA and protein expression, prostate cancer cell lines (LNCaP, DU145 and PC3) were stimulated with 1 nM somatostatin and 1 nM Octreotide for different periods of time. We found a time-dependent manner of up-regulation of *XAF1 *mRNA and protein in the cells treated with somatostatin and Octreotide (Figure 2, 3 and 4).

## Discussion

Most prostate tumours are initially androgen-dependent but become androgen-independent and eventually refractory to the hormone [5]. There are many regulative factors among its progression, relapse and tumour outgrowth. Prostate cancer cells evade apoptotic cell death by a variety of mechanisms [6,7]. *XAF1*, a potent apoptosis-inducer [8], plays a significant role in the process. A number of studies have shown that *XAF1 *can sensitize cancer cells to TRAIL, TNF-α, Fas, IFN-β and MEK inhibitor-induced apoptosis in vitro [12,26-29]. Moreover, some researchers have recently indicated the effect of *XAF1 *combination with these factors on inhibition of tumour growth in vivo and demonstrated that *XAF1 *can hinder tumour progression and promote outright regression in combination with TRAIL [30]. *XAF1 *mRNA is expressed at low or undetectable levels in most cancer cell lines, and transcriptional down-regulation in tumour cells as opposed to corresponding normal tissues and has been shown to occur at different frequencies in gastric adenocarcinomas, colorectal cancer, urothelial carcinomas, malignant melanomas, clear-cell renal cell carcinomas [8,13-16,31], non-small cell lung cancer, bladder cancer and B chronic lymphocytic leukemia [15,32,33].

Human prostate epithelial cells (RWPE-1) and prostate cancer cells (LNCaP, DU145 and PC3), which exhibit different features of prostate cancer progression from early stages to androgen independent stages, could mimic the development of prostate cancer clinically. Understanding the regulating effects of *XAF1 *during the whole progression may help us find potential therapeutic strategies for prostate cancer patients. To our knowledge, little is yet known about the regulatory effects of *XAF1 *in many different types of human cancers. Three prostate cancer cell lines LNCaP, DU145 and PC3 were well established in laboratory experiments. Their invasive characteristics were found to be different among the three cell lines: lower invasive ability of LNCaP, medium invasive ability for DU145 and a higher ability for PC3. The varying expression of *XAF1 *suggests a causal changing of androgen dependency and invasiveness in the development of prostate cancer.

The antiproliferative effect of somatostatin may result from increased apoptosis. In breast cancer cells MCF-7, the cytotoxic effect of somatostatin is dependent on SHP-1 and results from caspase 8 activation, cell acidification and mitochondrial dysfunction [34]. Apoptosis is induced by SSTR3 as a result of the induction of *p53 *and *Bax *[35] and is also induced by SSTR2 in HL-60 cells that express endogenous SSTR2 [36] and in human pancreatic cancer cells expressing mutated *p53 *and devoid of endogenous SSTR2, after correction of the deficiency by expression of SSTR2 [37]. Thus, somatostatin can induce apoptosis by *p53*-dependent and -independent mechanisms. SSTR2 induces apoptosis in a tyrosine phosphatase SHP-1-dependent manner.

Currently, several somatostatin analogues including Octreotide, Lanreotide, Vapreotide, Seglitide and so on, are available for the treatment of several kinds of disorders. Octreotide was the first developed analogue and is widely used for symptomatic treatment of hormone secreting neuroendocrine tumours. It has higher affinity for SSTR2 and shows significant anti-neoplastic actions in tumours expressing SSTR2 [38]. It remains the drug of choice for application in a majority of pure NE tumours because such tumours predominantly express SSTR2 [39]. However, other somatostatin analogues such as Lanreotide, which have good affinity for SSTR5 in addition to that for SSTR2, may advantageously recognize SSTR5 expressing tumours. But the relationship between *XAF1 *and somatostatin receptors needs further elucidation.

In our previous studies [24], we found that somatostatin up-regulated the expression of SSTR1-5, and that apoptosis was activated mainly via the induced expressions of SSTR2 and SSTR3. The effects of somatostatin on the prostate cancer cells may be mediated by enhanced expression of *XAF1 *through its pro-apoptotic effect. Somatostatin and Octreotide up-regulate *XAF1 *mRNA and protein in all prostate cancer cell lines, but the underlying mechanisms need further investigations. Likely, up-regulation of *XAF1 *mediated by somatostatin and Octreotide triggers cancer cell apoptosis.

## Conclusions

To our knowledge, little is known about the regulatory effects of *XAF1 *in many different types of human cancers. We report, the first time, that somatostatin and Octreotide up-regulate *XAF1 *mRNA and protein expression in LNCaP, DU145 and PC3 prostate cancer cell lines. Our findings suggest that *XAF1 *down-regulation may contribute to the prostate cancer development. The enhanced *XAF1 *expression by somatostatin indicates a promising strategy for prostate cancer therapy.

## Abbreviations List

NE: neuroendocrine; RWPE-1: human prostate epithelial cell line; SSTR: somatostatin receptor; *XAF1*: X-linked inhibitor of apoptosis protein-associated factor-1; *XIAP*: X-linked inhibitor of apoptosis.

## Competing interests

The authors declare that they have no competing interests.

## Authors' contributions

ZQX and ZLZ carried out experimental procedures and drafted manuscript. RY participated in its design. CDL and SN revised it critically. SLL and ZXL guaranteed the whole study. All authors read and approved the final manuscript.
